# Discrimination among Fresh, Frozen–Stored and Frozen–Thawed Beef Cuts by Hyperspectral Imaging

**DOI:** 10.3390/foods13070973

**Published:** 2024-03-22

**Authors:** Yuewen Yu, Wenliang Chen, Hanwen Zhang, Rong Liu, Chenxi Li

**Affiliations:** 1 State Key Laboratory of Precision Measuring Technology and Instruments, Tianjin University, Tianjin 300072, China; yuyuewenhappy@163.com (Y.Y.); chenwenliang@tju.edu.cn (W.C.); zhzhtjujy11@163.com (H.Z.); 2 School of Precision Instruments and Optoelectronics Engineering, Tianjin University, Tianjin 300072, China

**Keywords:** hyperspectral imaging system, beef cut, frozen–thawed, competitive adaptive reweighted sampling, classification

## Abstract

The detection of the storage state of frozen meat, especially meat frozen–thawed several times, has always been important for food safety inspections. Hyperspectral imaging (HSI) is widely applied to detect the freshness and quality of meat or meat products. This study investigated the feasibility of the low-cost HSI system, combined with the chemometrics method, to classify beef cuts among fresh (F), frozen–stored (F–S), frozen–thawed three times (F–T–3) and frozen–thawed five times (F–T–5). A compact, low-cost HSI system was designed and calibrated for beef sample measurement. The classification model was developed for meat analysis with a method to distinguish fat and muscle, a CARS algorithm to extract the optimal wavelength subset and three classifiers to identify each beef cut among different freezing processes. The results demonstrated that classification models based on feature variables extracted from differentiated tissue spectra achieved better performances, with ACCs of 92.75% for PLS-DA, 97.83% for SVM and 95.03% for BP-ANN. A visualization map was proposed to provide detailed information about the changes in freshness of beef cuts after freeze–thawing. Furthermore, this study demonstrated the potential of implementing a reasonably priced HSI system in the food industry.

## 1. Introduction

Meat and meat products contain essential nutrients and are thus important in the human diet and have a high economic value [1,2,3]. With the development of globalization, the demand for frozen meat products is increasing rapidly. During freezing and cold storage, fresh meat suffers sweeping deteriorations in both its physical properties and chemical constituents [4]. It has been reported that redness and water-holding capacity decrease, while yellowness and lipid oxidation obviously increase [5]. The freeze–thaw cycle may also cause some potential biosafety issues, such as microbial multiplication and the production of various biotoxins, which may arise in terms of food safety [6]. Due to their similar color and texture, it is difficult to differentiate fresh from frozen–thawed meat by eye. Consumer awareness of meat safety has been growing rapidly over the past few years [7,8,9]. Therefore, it is of great significance to develop a methodology for meat freshness and quality detection, especially for the classification of fresh, frozen–stored and frozen–thawed meat.

Traditional methods using either sensory or analytical methods, such as gas chromatography (GC), high-performance liquid chromatography (HPLC) [10], polymerase chain reaction (PCR), enzyme-linked immunosorbent assay (ELISA) [11] and biosensor, can provide reliable information about meat freshness and quality [12]. However, these methods are time-consuming, destructive and require sophisticated laboratory analysis. Recently, numerous studies have focused on developing rapid, non-destructive, low-cost and reliable techniques to detect meat quality and freshness [13]. Huang et al. [14] attempted to measure TVB-N content in pork meat by integrating near infrared spectroscopy, computer vision (CV) and electronic nose (E-nose) techniques. Romaniello Roberto et al. [15] developed a rapid method based on image analysis techniques to detect the presence of nitrates and nitrites in raw hams by inspecting the surface of a slide. Zhang et al. [16] utilized a hyperspectral imaging system to determine the texture parameters of Tan mutton. During frozen storage and thawing, the quality of meat is mainly affected by cell rupture, the oxidation of fat and the denaturation of myofibrillar and myoplasm proteins [5]. As the main components of meat are water, protein, fat and carbohydrate, the spectroscopic properties of these components are strongly related to these kinds of changes, which is of great interest for identifying fresh, frozen–stored and frozen–thawed meat [17]. 

Food safety is one of the biggest public issues occurring around the world. Microbiological, chemical and physical hazards can lead to food safety issues, which may occur at all stages of the supply chain. In order to tackle food safety issues and safeguard consumer health, rapid, accurate, specific, field-deployable detection methods that meet diverse requirements are among the imperative measures for food safety assurance [18]. With their advantages of being non-destructive, rapid and comparatively low-cost, spectroscopic techniques, such as ultraviolet–visible (UV-VIS), near infrared spectroscopy (NIR) [19,20], mid-infrared (MIR) spectroscopy and raman spectroscopy [21], are widely applied in meat detection on the basis of spectral analysis [22]. Thyholt and Isaksson [23] utilized NIR diffuse reflectance spectroscopy in combination with K-nearest neighbor (KNN) to classify frozen–thawed beef. Cáceres-Nevado et al. [24] developed Fourier transform infrared (FT-NIR) spectroscopy combined with partial least squares discriminant analysis (PLS-DA) to distinguish fresh and frozen–thawed pig loins, achieving an accuracy of 98.21%. However, most spectroscopic techniques collect single-point spectra of samples using an integrating sphere or fiber optic probe. This may lead to unrepresentative spectral sampling and false positive results due to heterogeneity and high dependency on the composition changes of different tissues. 

Hyperspectral imaging (HSI), which can acquire spectral and spatial information at the same time, has emerged as one of the most powerful techniques for detecting meat quality and freshness [25,26]. With two spatial dimensions and one spectral dimension, the hypercube provides information about the structure and composition of heterogeneous meat samples. Cheng, J. et al. [27] evaluated the feasibility of non-destructive detection of carbonyl and total sulfhydryl contents by fluorescence HSI to visualize the protein oxidation degree of pork during the freeze–thaw process. The spectral analysis method is also important for HSI applications. Pu et al. [28] used HSI combined with a convolutional neural network (CNN) to distinguish between fresh and frozen–thawed beef samples. The CNN model, using early feature fusion of spectra and texture, showed excellent results, with an accuracy of 88.89%. To evaluate the protein content of various processed pork samples, Ma et al. [29] selected the best spectral profile from reflectance, absorbance and Kubelka-Munk (K-M) spectra by comparing the performances of their BP-NN models. Cheng et al. [30] investigated the possibility of using HSI in the spectral range of 1000–2200 nm to characterize myofibrils cold structural deformation degrees of frozen pork samples. A spectral angle mapping (SAM) algorithm was used to extract spectral information for regression. The optimized PLSR models based on the spectral angles calculated by the SAM algorithm achieved better performance. 

The general challenge with HSI application is to achieve high quality and sensitivity without increasing the cost and complexity of the instruments [31,32]. There is a demand for a low-cost, portable HSI system for meat quality assessment. However, the freshness attributes at different locations of the meat sample may be diverse, whereas the spectral resolution, imaging range and resolution are important for discriminating the freshness and freeze–thaw state of the meat. Because the high data curse of dimensionality also makes the HSI classification model more complex, systematic research into spectral features and model optimization can contribute to accurate freeze–thaw meat identification [33]. 

This study aimed to investigate the feasibility of a low-cost HSI system combined with a chemometrics method to classify fresh (F), frozen–stored (F–S), frozen–thawed three times (F–T–3) and frozen–thawed five times (F–T–5) beef cuts. To meet the demand for rapid on-site detection, a compact, low-cost and portable HSI system was designed and calibrated for meat measurement. By comparing the spectral characteristics of F, F–S, F–T–3 and F–T–5 beef samples, the classification model was developed for meat analysis with a method to distinguish fat and muscle, a CARS algorithm to extract the optimal wavelength subset, and a classifier to identify the beef cut among different freezing processes. Then, the performance of the classification models was evaluated, and a visualization map was proposed to provide detailed information about the quality changes of beef cuts after different freeze–thaw processes. This study also highlighted the potential for low-cost, high-resolution spectral imaging instruments to support food safety testing as well as other research and applications. 

## 2. Materials and Methods

### 2.1. HSI System and Image Acquisition

**Figure 1:**
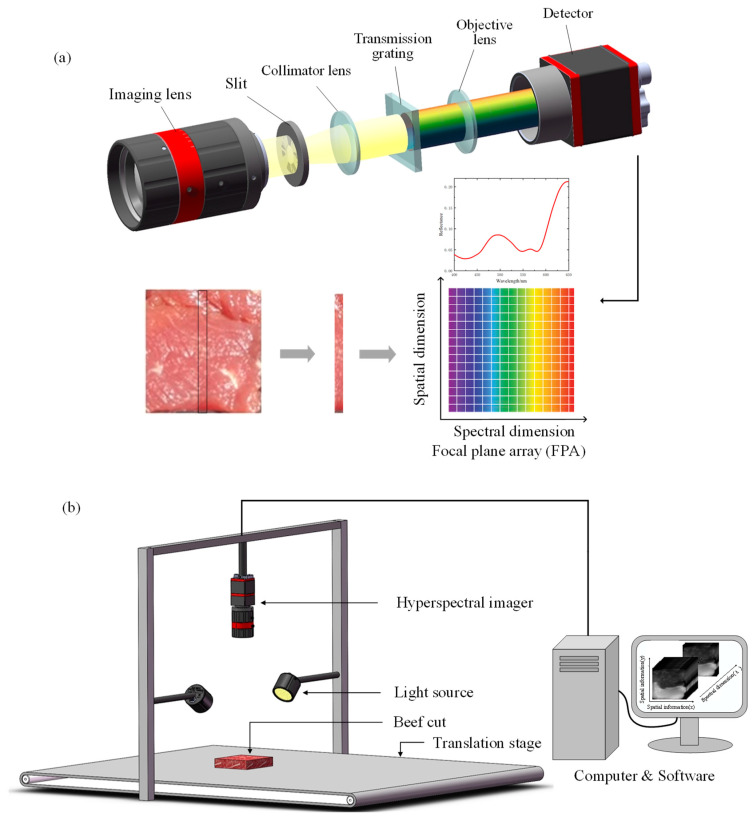
Schematic diagram of the main components. (**a**) The hyperspectral imaging system; (**b**) The hyperspectral image acquisition system.

In this study, we designed a compact, low-cost, portable push-broom hyperspectral imaging (HSI) system with a price range of USD 700 to USD 1100 and a spectral range from visible to near-infrared. As shown in Figure 1a, the developed HSI system consists of an imaging lens (focal length, FL = 16 mm), a high-precision slit (25 μm × 3 mm), a collimating lens (FL = 30 mm), a transmission grating (groove density = 600 lines/mm, dimensions = 25 × 25 mm^2^), an objective lens (FL = 25 mm) and a detector (acA2440-75 μm, Basler., Mannheim, Germany) with a two-dimensional focal plane array (FPA) to capture the spectrograms. The FPA records spatial information in one dimension (x) and spectral information in the other dimension (λ). To meet the requirement of low cost, the HSI system housing was 3D printed and assembled with commercial off-the-shelf components. The hyperspectral data were acquired with the samples placed on a translation stage (PA200-ST242-C-SR, ZOLIX, Beijing, China) and illuminated with halogen lamps, as shown in Figure 1b. The HSI system was controlled by software developed in LabVIEW 2018 (National Instruments), which could control the camera exposure time, motor speed and binning mode, and select the wavelength range. 

### 2.2. HSI System Calibration

The spectral response of the HSI system was calibrated using a monochromator (Newport Corporation, Irvine, CA, USA) in the range of 400–800 nm. To perform the wavelength calibration, a linear regression analysis was conducted between the pixel numbers of the λ-dimension in the FPA and the wavelength peaks of the monochromatic light. The average value of the intensity in the λ-dimension was taken as the ordinate and the number of pixels as the abscissa to fit the pixel position–wavelength equation, which could be used to calculate the wavelengths of the spectral channel on the FPA directly. The fitting result showed a good relationship with $R^{2}$ = 0.99, and the fitting equation can be expressed as

(1) $$\lambda_{i}=\left\{\begin{array}{c} -7.37e^{-06}i^{2}-0.201i+803.26\;\;\;\;\;50<i\leq 1000\\ -2.09e^{-06}i^{2}-0.213i+809.19\;\;\;\;\;1000<i\leq 1940 \end{array}\right.$$

where i is the number of pixels and $\lambda_{i}$ is the central wavelength of i. According to the spectral response calibration results, the original hyperspectral data can be further processed into monochromatic images of different spectral bands. 

**Figure 2:**
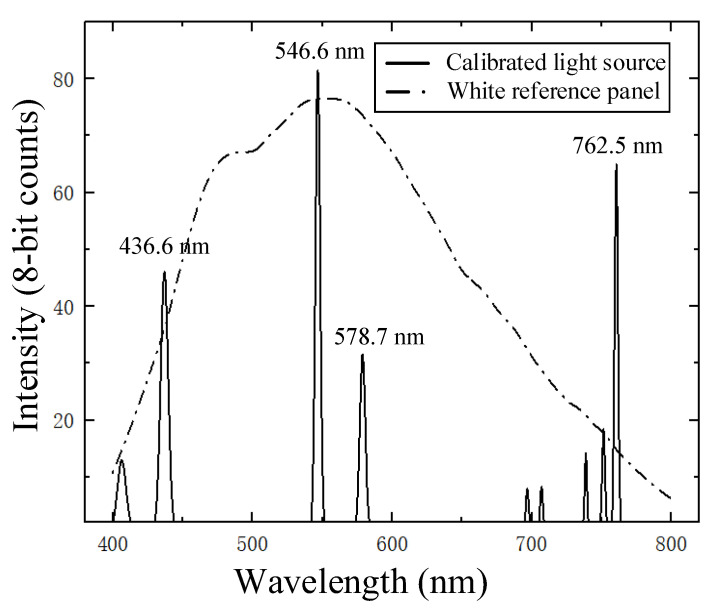
HSI calibration data. Solid lines show spectral calibration peaks from the calibrated light source. The dotted line is the measured spectrum from the reference standard with 30% reflectance.

The spectral resolution of the HSI system was evaluated with a mercury–argon calibration source. The spectral resolution, measured as the full width at half maxima (FWHM), was calculated from the spectral calibration data shown in Figure 2. The results indicated that the mercury peak at 546.6 nm had a minimum FWHM of 3.6 nm. The difference between the spectral calibration results and the characteristic spectral lines was less than 1 nm within the range of 400–800 nm. 

### 2.3. Sample Preparation

Fresh beef tenderloin was purchased from three local markets in Tianjin, China, then transported to the laboratory within 30 min and cut into a total of 240 pieces with similar sizes of 3 (±0.5) cm × 3 (±0.5) cm × 0.5 (±0.1) cm (length × width × thickness). Among these samples, the first group of 60 was used as fresh (F) samples for further handling. The second group of 60 samples, defined as F–S samples, was stored at −18 °C for 24 h. In this study, one freeze–thaw cycle was defined as the sample having been frozen at −18 °C for 24 h and then thawed at room temperature (25 °C) for 4 h. The third group of 60 samples, defined as F–T–3, was frozen and thawed three times. The fourth group of 60 samples, defined as F–T–5, was frozen and thawed five times. During freezing and thawing, all samples were vacuum-packed to avoid surface drying or external contamination. The above experiments were designed to simulate the frozen–stored and frozen–thawed states that frequently occur in meat storage and shelf life. 

### 2.4. Classification Model

**Figure 3:**
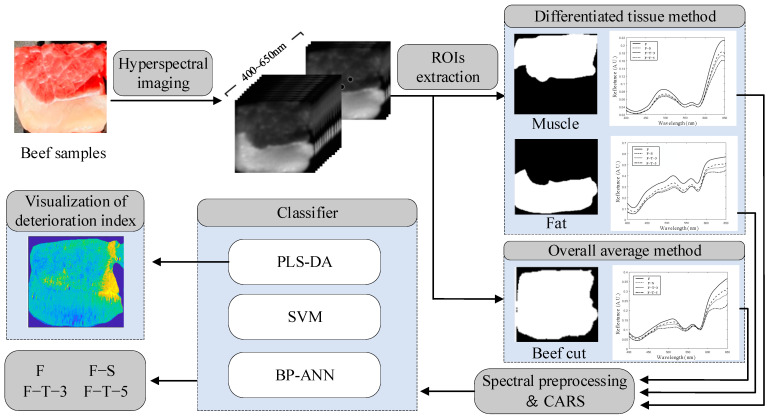
Flowchart of the main steps of the classification model.

In this study, the F, F–S, F–T–3 and F–T–5 samples were classified with models based on differentiated tissue and feature extraction methods. The flowchart of the main steps of the classification model is depicted in Figure 3. Based on their spectral characteristics, the regions of interest (ROIs) of fat, muscle and beef cut were distinguished by comparing the Euclidean distance between their spectra. The spectral features of fat and muscle were extracted using competitive adaptive reweighted sampling (CARS). Then, partial least squares discriminant analysis (PLS-DA), support vector machine (SVM) and BP artificial neural network (BP-ANN) were applied to establish the model for classifying F, F–S, F–T–3 and F–T–5 samples. The deterioration index was determined and visualized to provide detailed information about the quality changes of each beef cut after F–S and frozen–thawed (F–T) processing. Data handling and analysis were performed using MATLAB software version R2022a (MathWorks, Natick, MA, USA). 

#### 2.4.1. HSI Processing

To improve the quality of the hyperspectral images, the original images ($I_{0}$) were calibrated with black and white standard images, according to the following equation [34]:

(2) $$I=\frac{I_{0}-B}{W-B}\times 30\%$$

where B is the black reference image obtained by covering the camera with an opaque cap (~0% reflectance), and W is the white reference image obtained under the same condition as the beef samples were scanned, but for a whiteboard with a uniform, stable reflectivity (about 30% reflectance). 

After the hyperspectral images were calibrated, segmentation was performed to distinguish the fat and muscle tissues of the beef cuts. First, the standard spectra of fat and muscle were collected using a spectrometer. Then, the similarity between the spectral data x of each pixel p(i,j) in the hyperspectral image and the standard spectrum was calculated using the Euclidean distance, as follows:

(3) $$D_{m}=\sqrt{\sum_{i=1}^{n}{\left(x-u_{m}\right)}^{2}}$$

(4) $$D_{f}=\sqrt{\sum_{i=1}^{n}{\left(x-u_{f}\right)}^{2}}$$

where n is the spectral feature dimension, $u_{m}$ is the standard spectrum of muscle, $u_{f}$ is the standard spectrum of fat, $D_{m}$ is the similarity between the spectral data x and $u_{m}$ and $D_{f}$ is the similarity between spectral data x and $u_{f}$. If $D_{m}$ was greater than or equal to $D_{f}$, the pixel p(i,j) was labeled as muscle. Otherwise, it was labeled as fat. The ROIs of fat and muscle were thus extracted.

To reduce the influence of noise, scattering and baseline drift, the spectral data were preprocessed with a Savitzky–Golay smoothing filter (SG), the first derivative (FD), the standard normal variate (SNV) and the detrending algorithm. The FD effectively removes interference caused by baseline drift or a flat background, while the SNV eliminates surface scatter, as well as the effect of light-range changes on the diffuse reflectance spectrum. Detrending was used to remove baseline drift. 

The hyperspectral data are high-dimensional and co-linear, with two spatial dimensions and one spectral dimension, making the classification model more complex and easily overfitting. Then, feature extraction is required to obtain the relevant information to make the classification model more accurate and robust. In this study, CARS was applied to select key wavelengths of the hyperspectral data [35]. Based on the principle of “survival of the fittest”, CARS evaluates the optimal combination of the wavelengths existing in the full spectrum, coupled with partial least squares regression. 

#### 2.4.2. Classification Model

Classification models play an important role in the discrimination of F, F–S, F–T–3 and F–T–5 beef samples. In this study, both linear and nonlinear algorithms were applied to create sample classification models. The feasibility of these models was explored using cross-validation and prediction tests. 

PLS-DA, a linear discriminant method based on partial least squares regression (PLSR), was used to correlate the spectra (X) and the response variable (Y) by searching the latent variables (LVs) to maximize the covariance between X and Y, as follows [36]:

(5)  Y=Xb+e

where b is the regression coefficient matrix and e is the residual information matrix. Unlike quantitative analysis, the Y variables used in qualitative analysis were dummy variables assigned for the different classes ‘F’, ‘F–S’, ‘F–T–3’ and ‘F–T–5’ as 1, 2, 3 and 4, respectively. The 10 optimal LVs were selected based on the minimum value of the cross-validation root mean square error (RMSECV). The error threshold was set to be ± 0.5 for class assignment.

SVM is a kernel-based machine-learning technique that has been widely used in discriminant analysis. It aims to find a linear separating hyperplane such that the distance of all samples from that hyperplane is maximized. SVM has a good generalization capability with the kernel function that maps the input vector x to a higher-dimensional feature space [37]. In this study, the radial basis function (RBF) was employed as the kernel function. The classes {F, F–S, F–T–3, F–T–5} were represented as (0, 0, 0, 1), (0, 0, 1, 0), (0, 1, 0, 0), and (1, 0, 0, 0), respectively. The cross-validation was utilized to achieve optimal (C, γ) as (0.18, 0.008).

BP-ANN is a kind of feed-forward neural network that can work without prior knowledge and human effort to preprocess raw data [38]. It is composed of three layers: input layer, hidden layer and output layer. The tansig function is widely used in the transfer function between the input layer and the hidden layer, and the purelin function is used between the hidden layer and the output layer. The input layer has the same number of input nodes as the number of spectral features. The performance can be improved by changing the number of hidden layer nodes.

#### 2.4.3. Model Evaluation and Visualization

Before establishing the model, the datasets were divided into training and prediction sets (75% and 25% of the total samples, respectively) using sample set partitioning based on joint x-y distances (SPXY). SPXY employs a partitioning algorithm that takes into account the variability in both spectral data (x) and classification categories (y). In this manner, the multidimensional space may be covered more effectively than in partitioning schemes based on x-information alone (such as the Kennard–Stone (KS) algorithm) or random sampling (RS) [39]. Samples in the training set were used to establish the classification model. A hierarchical 10-fold cross-validation was used on the training set to verify the performance of the model. Of the 10 subsamples, a single subsample was retained as the validation data for testing the model, and the remaining 9 subsamples were used as training data. The cross-validation process was then repeated 10 times (the folds) with every subsample used exactly once as the validation data. For classification problems, this method can make each fold contain roughly the same proportions of class labels. 

The performance of the classification models was evaluated in terms of accuracy (ACC), sensitivity (SEN) and specificity (SPEC), which were calculated as follows:

(6) $$Accuracy=\frac{TP+TN}{TP+TN+FP+FN}\;$$

(7) $$Sensitivity=\frac{TP}{TP+FN}$$

(8) $$Specificity=\frac{TN}{TN+FP}$$

where TP, TN, FN and FP are the true positive, the true negative, the false negative and the false positive, respectively. 

Based on these metrics, the confusion matrices were also calculated to explore the detailed classification performance. The confusion matrix is a concept from machine learning that contains information about actual and predicted classifications performed by a classification system. A confusion matrix has two dimensions, one indexed by the actual class of an object and the other by the class that the classifier predicts [40]. Sensitivity and specificity can be calculated using the confusion matrix. Since hyperspectral imaging obtains spectral information about the beef cut at each pixel, the deterioration level can be predicted. In this study, the deterioration index was calculated according to Equation (5). The visualization map was generated using pseudocolor to render the predicted deterioration index of each pixel. 

## 3. Results

### 3.1. Spectral Analysis of Fresh, Frozen-Stored and Frozen-Thawed Samples

**Figure 4:**
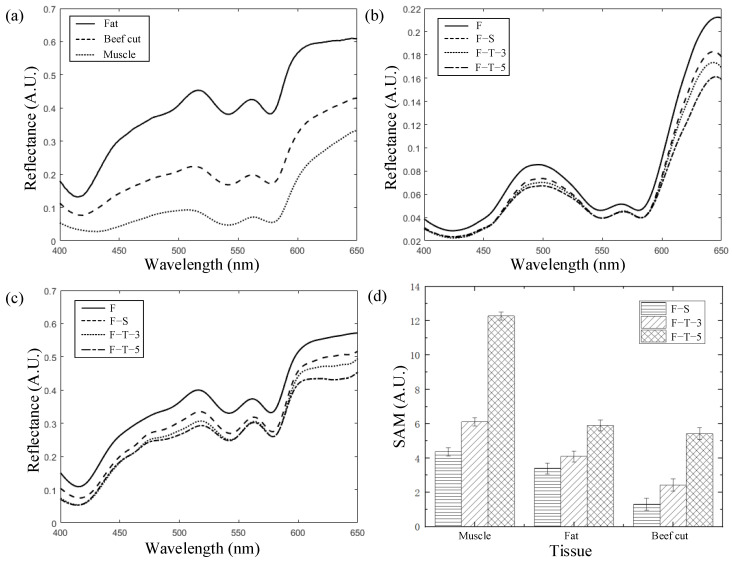
The reflectance spectra of different beef tissues among F, F–S, F–T–3 and F–T–5. (**a**) The reflectance spectra of muscle, fat and beef cut; (**b**) The reflectance spectra of F, F–S, F–T–3, F–T–5 fat; (**c**) The reflectance spectra of F, F–S, F–T–3, F–T–5 muscle; (**d**) Spectral angle mapper for F, F–S, F–T–3 and F–T–5 among different tissues.

The reflectance spectra of different beef tissues among F, F–S, F–T–3 and F–T–5 are illustrated in Figure 4. The spectral characteristics observed in the range of 400–650 nm are related to overtones and the combinations of the fundamental vibrations of the C-H, N-H, O-H and S-H functional groups. The mean spectra of muscle, fat and beef cut were compared, as shown in Figure 4a. Primary peaks near 420 nm, 550 nm and 572 nm were observed, apparently owing to the absorption bands of respiratory pigments, specifically hemoglobin (Hb) and myoglobin (Mb), which may also influence the color of meat [41,42]. The reflectance of fat is much higher than that of muscle because fatty tissue contains fewer pigments [43,44].

As shown in Figure 4b,c, the mean spectral reflectance of fresh muscle and fat are the highest, followed by the F–S, F–T–3 and F–T–5 samples. As high reflectance values usually indicate high amounts of water, the decrease is possibly due to dehydration and water loss during frozen–stored and frozen–thawed processing. Furthermore, the two bands appearing near 500 nm and 650 nm are related to the oxidation of Hb or Mb [45,46]. During F–S and F–T processing, temperature fluctuations or discontinuities can induce an increase in lipid peroxides, thiobarbituric acid reactive substances (TBARS) and carbonyl content [47,48]. Additionally, Hb and Mb are gradually oxidized to methemoglobin (metHb) and metmyoglobin (metMb), which may lead to surface discoloration [49].

Since the reflectance spectra of muscle and fat vary with different processing methods, a spectral angle mapper (SAM) was employed to quantize the difference between the spectra [50]. As shown in Figure 4d, the average values of the SAM among the F, F–S, F–T–3 and F–T–5 muscle samples are 4.36, 6.11 and 12.27, respectively, indicating high variations among each group of samples. The SAMs of both fat and muscle are significantly higher than those of the beef cut, indicating that the spectral characteristics of fat and muscle are more pronounced than those of the beef cut. As the muscle and fat change differently with F–S and F–T processing, the classification model based on differentiated tissue may be more effective than the model based on overall averaging.

### 3.2. Feature Wavelength Selection

**Figure 5:**
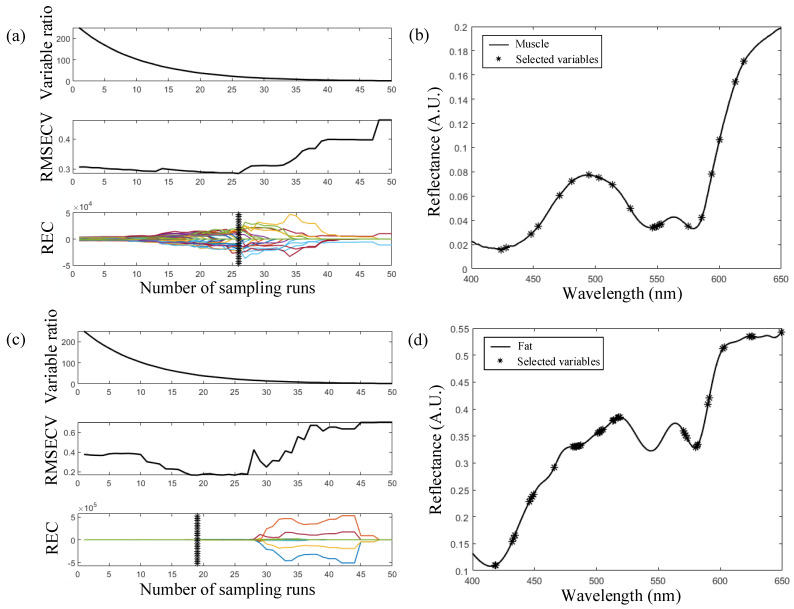
The characteristic selection by CARS. (**a**) Characteristic selection process of muscle; (**b**) Characteristic selection results of muscle; (**c**) Characteristic selection process of fat; (**d**) Characteristic selection results of fat.

Determining the feature wavelengths from HSI data is very important for achieving better classification performance, because similar or close wavelengths carry the same or similar information [51]. In this study, CARS was used to select feature wavelengths from the raw HSI data for fat and muscle. The characteristic wavelength screening process for muscle and fat is shown in Figure 5a,c. The Variable Ratio, RMSECV and REC show the changing trend of the number of variables sampled, the RMSECV values and regression coefficients (REC) for each variable with the increasing of samples runs, respectively [35]. The wavelength subset corresponding to the minimum RMSECV (indicated by ‘*’) was selected as the characteristic variable. Therefore, 21 variables were selected from the spectrum of fat, as shown in Figure 5b, and 42 were selected from the spectrum of muscle, as shown in Figure 5d. The optimal variables are mainly concentrated near the bands of 420 nm, 500 nm, 550 nm, 572 nm and 650 nm, indicating that the F–S and F–T processes induce redox reactions of the Hb and Mb of beef samples. 

### 3.3. Classification Results

The performance of three classifiers with different preprocessing algorithms was evaluated with the ACC metric, as shown in Table 1. Generally, the classification models based on differentiated tissue achieved better accuracy than the overall average of the beef cut because the muscle and fat tissue change differently during the F–S and F–T processing. There was not much difference between the two nonlinear classifiers with input variables selected by CARS. The PLS-DA, SVM and BP-ANN classifiers achieved prediction accuracies of 92.75%, 97.83% and 95.03%, respectively. The results indicate that distinguishing the types of tissue has a strong discriminative ability for F, F–S, F–T–3 and F–T–5 beef samples.

It was also crucial to select characteristic wavelengths for classification model establishment. When the raw spectra were used as the input of the classification models, the average ACCs were lower than 86%. However, by reducing the number of variables from 251 to 21 and 42, the ACCs increased significantly by 18.79% and 17.2% for SVM and BP-ANN, respectively. Preprocessing can also improve the performance of the classification model by effectively eliminating baseline drift and background interference [52]. In addition, compared with other classifiers, the SVM classifier showed its own powerful capability in improving prediction accuracy and model performance. This may be due to the limited ability of the linear method PLS-DA to extract complex nonlinear features from original spectra, while the nonlinear method BP-ANN is prone to local minimization [53]. 

**Figure 6:**
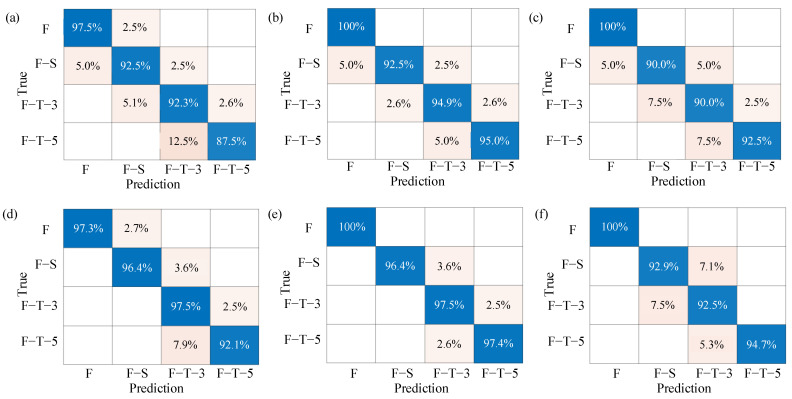
Confusion matrix detailing the multiclass discrimination results based on CARS. (**a**) PLS-DA based on overall average; (**b**) SVM based on overall average; (**c**) BP-ANN based on overall average; (**d**) PLS-DA based on differentiated tissue; (**e**) SVM based on differentiated tissue; (**f**) BP-ANN based on differentiated tissue.

The confusion matrix, calculated using sensitivity and specificity metrics, provided insights into the classification performance of the models. As shown in Figure 6, the correctly classified results were located on the diagonal, indicating the sensitivity of the model. As shown in Figure 6a–c, the SVM and BP-ANN classifiers based on the overall average of beef cut could identify 100% of fresh beef, while the sensitivity of PLS-DA to the F group is 97%, higher than that of the F–S and F–T groups. This result can be attributed to the fact that fresh beef maintains its moisture content and biochemical integrity, resulting in distinct spectral or textural patterns that are easily captured by the classifiers [54]. During freezing and thawing processing, freezing causes the formation of ice crystals within the muscle tissue, leading to moisture loss and cell disruption [55]. Thawing may result in further degradation of the meat’s structure and biochemical composition [56]. These physicochemical changes result in significant differences in spectral and textural characteristics between fresh and frozen–thawed meat. Further analysis also found that most of the misclassified samples contained a large area of fat, which disturbed the classification results due to its distinct light-absorption and -scattering properties compared to muscle tissue. 

The confusion matrices of the three classifiers based on differentiated tissue are shown in Figure 6d–f. Compared with the model based on the overall average of the beef cut, the sensitivity of the three classifiers based on differentiated tissue was significantly improved. The number of misclassifications between adjacent classes has decreased significantly, as the sensitivity of SVM for F–S, F–T–3 and F–T–5 reached more than 96%. The results demonstrate that the differentiated tissue method can efficiently improve the accuracy of the classification model. 

### 3.4. Visualization of the Deterioration Index

**Figure 7:**
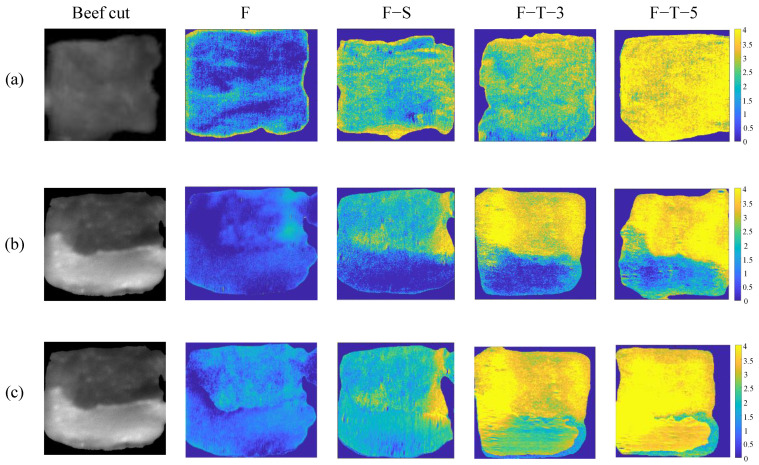
Visualization of F, F−S, F−T−3 and F−T−5 beef cut classification maps based on (**a**) and (**b**) Overall average method and (**c**) Differentiated tissue method.

The visualization map provides detailed information about the changes in freshness of beef cuts after F–S and F–T processing. In this study, the PLS-DA model combining the differentiated tissue method with CARS was used to produce visualization maps on which the spatial distribution of the deterioration index is presented, as shown in Figure 7. Guided by the color scale derived from MATLAB’s “parula” colormap, the changes in freshness were evaluated by color variations. Specifically, the transition from blue to yellow indicated a deterioration index from low (F) to high (F–T–5). The background was artificially set to a dark blue color to avoid any confusing information. As shown in Figure 7a, with F–S and F–T processing, the color changes from blue to yellow, reflecting the trend of deterioration. However, for individual samples, the deterioration index varied with different tissues due to the heterogeneity of the beef cut. As shown in Figure 7b, with F–S and F–T processing, most of the fatty tissue was misclassified as fresh by the classification model based on overall average of beef cut. 

Most importantly, in this study, the fat and muscle were first segmented to establish discrimination models based on spectral features. As shown in Figure 7c, most of the fat tissue was correctly classified compared with the same region in Figure 7b. The above results indicate that the classification model performed better based on the segmentation of different types of tissue. The proposed classification model can accurately identify heterogenous beef samples with different freeze–thaw cycles, which helps in the accurate analysis of the changes in freshness of beef cuts. Hyperspectral imaging has provided an interesting approach to visualizing the distribution of freshness that cannot be achieved by the single-point spectrum.

Recently, more portable HSI instruments have been developed to meet the increasing demand for rapid food analysis. This study utilized portable equipment with a simplified design and 3D printed assembly, providing the potential to develop low-cost HSI for many online detection tasks with low budgets. There is also a large potential for combining an HSI system with chemometrics for applications in different matrices, such as food authenticity and adulteration. 

## 4. Discussion

This study was conducted to investigate the feasibility of HSI for classifying F, F–S, F–T–3 and F–T–5 beef. A compact HSI system was constructed to acquire the hyperspectral data of beef cuts. By extracting the feature variables, the ACCs of SVM and BP-ANN were significantly improved by 18.79% and 17.2%, respectively. Moreover, better results were acquired with ACCs of 92.75% for PLS-DA, 97.83% for SVM and 95.03% for BP-ANN classification models based on differentiated tissue spectra, as compared with ACCs of 90.35% for PLS-DA, 97.83% for SVM and 93.13% for BP-ANN classification models based on the overall average of beef cut. The deterioration index was determined and visualized to provide detailed information about the freshness changes of beef cuts after F–S and F–T processing.

This study established a discrimination model for meat analysis using differentiated tissue and feature extraction methods. In contrast to studies using spectroscopic techniques [24], this study employed hyperspectral imaging to detect heterogeneous beef samples and to generate visualization maps that clearly show the spatial distribution of the deterioration index. Currently, the majority of hyperspectral imaging-based meat detection methods rely on threshold segmentation to separate muscle and fat tissues and subsequently establish classification models using only muscle tissue spectra [57,58]. However, this approach overlooks the quality changes in fat tissue. This study utilized a tissue differentiation approach to comprehensively evaluate meat quality, significantly enhancing classification accuracy compared to the method of overall average. The low-cost, compact HSI system developed in this study could be profitably implemented in the food industry to provide consumers with commodities of high quality and reasonable prices. Moreover, HSI offers distinct advantages over other techniques in terms of speed, sample processing and manpower requirements. Further research should focus on exploring more effective methods for date acquisition and classification model establishment in future industrial applications. 

Requirements for sustainable food production are challengingly increasing in this area, with the growth of the world population and the globalization of the food market. In this context, food security, traceability and authenticity assurance become of utmost importance to guarantee consumers’ safety. Analytical approaches based on Green Analytical Chemistry (GAC) have contributed to food safety and quality assessment, as well as to food bioactivity studies [59,60]. The portable HSI system and frozen–thawed beef detection process introduced in this study avoid sample treatment and reagent use and can be used for multianalyte and multiparameter online detection, in line with the principles of GAC [61,62]. The integration of HSI technology with GAC principles holds significant importance for promoting transformation in the food industry. It will also provide a non-destructive, safe and environmentally friendly detection method for environmental protection, agriculture, industrial production, military reconnaissance and other fields. 

## Figures and Tables

**Table 1:** ACCs of the classifiers based on the overall average of beef cut and differentiated tissue.

	Input	No. of Wavelength	Accuracy (%) of Prediction		
			PLS-DA	SVM	BP-ANN
Overall average	RAW	251	83.75	76.25	75.00
	SG+FD	250	90.63	94.38	91.88
	CARS	21	90.35	95.63	93.13
Differentiated tissue	RAW	251	85.97	79.04	77.83
	SG+FD	250	92.11	95.63	93. 35
	CARS	21 (muscle) 42 (fat)	92.75	97.83	95.03

## Data Availability

The original contributions presented in the study are included in the article, further inquiries can be directed to the corresponding authors.
